# Influence of Composite Structure on Temperature Distribution—An Analysis Using the Finite Difference Method

**DOI:** 10.3390/ma16145193

**Published:** 2023-07-24

**Authors:** Ewelina Kubacka, Piotr Ostrowski

**Affiliations:** Department of Structural Mechanics, Łódź University of Technology, 93-590 Łódź, Poland; piotr.ostrowski@p.lodz.pl

**Keywords:** heat transfer, composite, periodic structure, biperiodic structure, finite difference method

## Abstract

Among composites, we can distinguish periodic structures, biperiodic structures, and structures with a functional gradation of material properties made of two or more materials. The selection of the composite’s constituent materials and the way they are distributed affects the weight of the composite, its strength, and other properties, as well as the way it conducts heat. This work is about studying the temperature distribution in composites, depending on the type of component material and its location. For this purpose, the Tolerance Averaging Technique and the Finite Difference Method were used. Differential equations describing heat conduction phenomena were obtained using the Tolerance Averaging Technique, while the Finite Difference Method was used to solve them. In terms of results, temperature distribution plots were produced showing the effect of the structure of the composite on the heat transfer properties.

## 1. Introduction

Composite materials are materials with specific designed properties. These properties can be obtained by combining certain materials in the relevant way. Composite structures can be characterized by better strength, resistance, or thermal conductivity than their individual component materials. In periodic and biperiodic structures or in those with a functional gradation of properties, it is possible to separate mentally repeatable cells with the same structure. The dimension of such a cell is called the microstructure parameter. Methods that are commonly used in such considerations are certainly the Finite Element Method [1,2]; asymptotic homogenization [3]; the variation of homogenization, introducing additional unknowns called microlocal parameters [4,5]; the higher order theory [6,7]; and the boundary element method [8]. Not all of them take into account the microstructure parameter, in contrast to the Tolerance Averaging Technique, which is used in this work [9,10]. This method is used in the analysis of a variety of structures, such as beams [11,12], plates [13] and shells [14,15]. The main reason for utilizing the Tolerance Averaging Technique in this study was the great alignment of its results to the exact solution when it comes to the stationary problem of heat conduction [16].

The Tolerance Averaging Technique makes it possible to average out the discontinuous coefficients in the heat conduction equation, which result from the different properties of the composite’s constituent materials. The resulting averaged equations, describing the heat flow in the composite structures, were solved numerically using the Finite Difference Method, which was implemented by the authors into MAPLE 2019 software. The proposed FDM algorithm is universal in terms of the dimensions of the composite, its structure, and the type of constituent materials, but not in terms of the assumed boundary conditions of thermal loads, because the number of equations of the Finite Difference Method depends on the number of nodes wherein the sought unknown function is not predetermined.

## 2. Various Composite Structures of Interest

**Figure 1:**
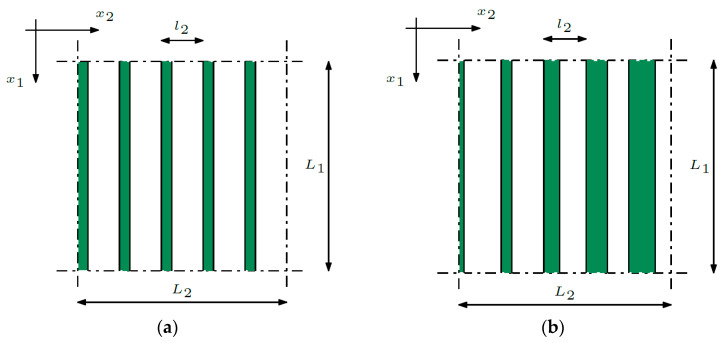
Composite with (**a**) periodic structure; (**b**) functionally graded structure.

Composite materials are often used in the design of plate and shell structures. Three different structures of this type were analyzed. The first structure is a periodic composite, as shown in Figure 1a. The dimensions of the composite are denoted by *L*_1_ and *L*_2_ and the material properties change recurrently along the *x*_2_-direction.

The dimension of the mentally separated cell, made of two materials, is denoted by *l*_2_. The volume proportion of individual materials in the cell is constant. The volume proportion of the first material is denoted by *v*_1,_ and the volume share of the second material is denoted by *v*_2_, while *v*_1_ + *v*_2_ = 1.

The second structure is a structure with a functional gradation of properties and is shown in Figure 1b.

The volume proportion of individual materials in the cell is not constant and is determined by a function that depends on the *x*_2_-coordinate. Therefore, the volume proportion of the first material is denoted by *v*_1_(*x*_2_), and the volume share of the second material is denoted by *v*_2_(*x*_2_).

**Figure 2:**
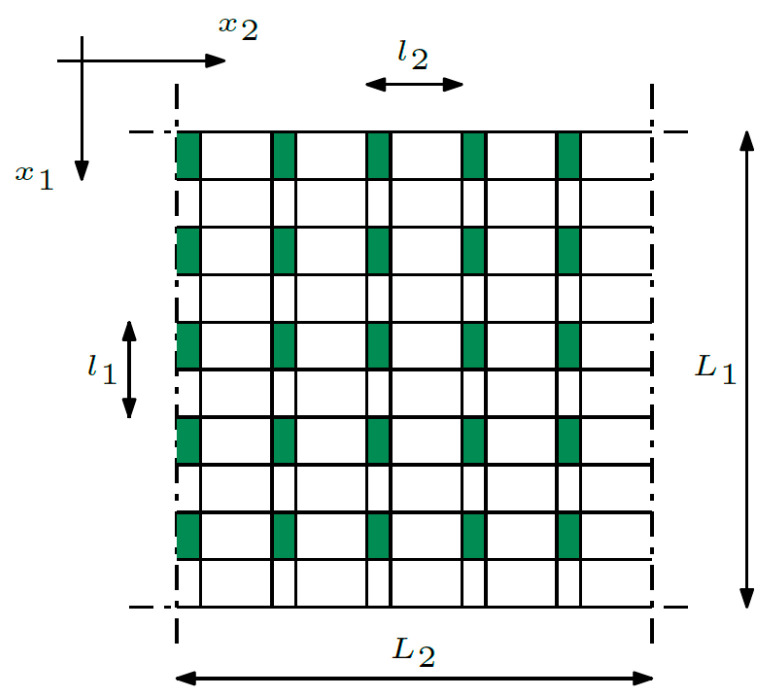
Biperiodic structure.

The last one—the third composite—is a biperiodic structure (refer to Figure 2). In the case of this structure, the properties change recurrently along both directions—*x*_1_ and *x*_2_. The dimensions of the mentally separated cell are denoted by *l*_1_ and *l*_2_.

The dimension of the cell, called the microstructure parameter, is dependent on the number of composite cells *N*.

## 3. Tolerance Averaging Technique

The Tolerance Averaging Technique, mentioned previously, was used to average Equation (1) of the heat transfer issue [17] to replace the terms with discontinuous coefficients:

(1) $$\partial_{i}\left(K_{ij}\partial_{j}\theta \right)-c\rho \;\dot{\theta }=0$$

where θ symbolizes the total temperature field; the components of the tensor of conductivity are denoted by *k_ij_* (*i*,*j* = 1, 2, 3), a specific heat by *c* and a mass density by ρ. Each cell is composed of two (periodicity) or four (biperiodicity) sub-cells, and the above-mentioned material properties within a given sub-cell are constant.

**Figure 3:**
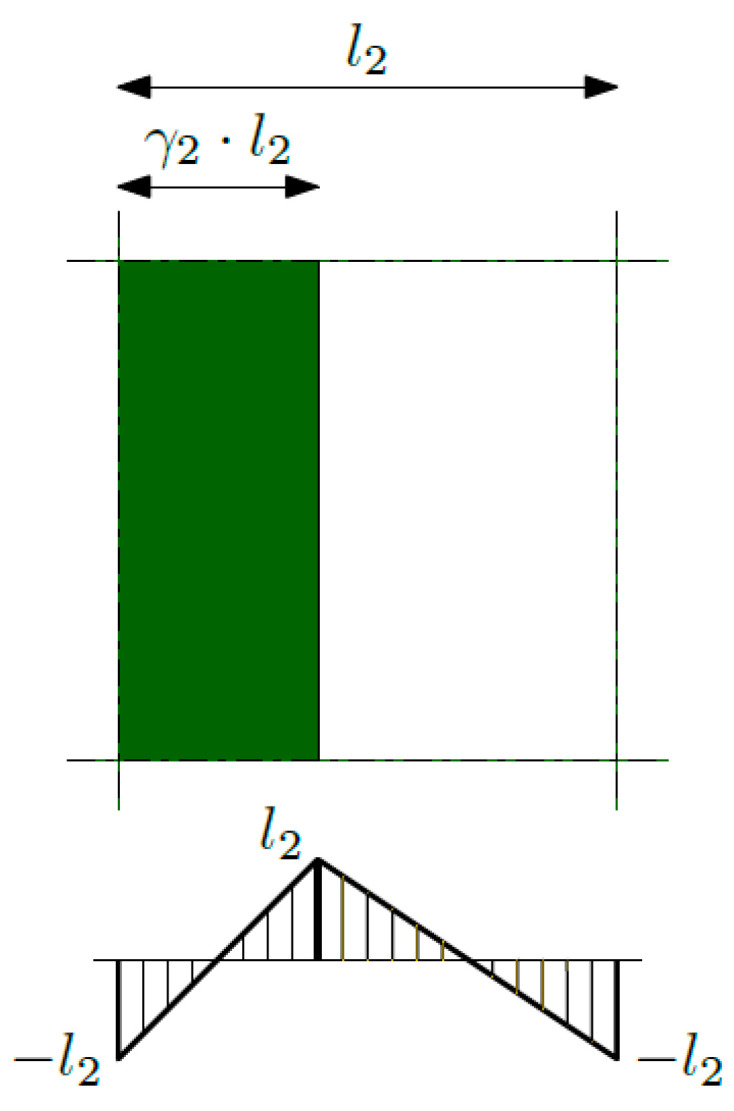
Fluctuation shape function *g*_1_.

The technique, used to average the discontinuous coefficients in Equation (1), is constantly developed and used in the analysis of periodic [18,19,20,21,22,23], biperiodic [24,25,26] and functionally graded structures [27,28]. The most relevant assumption of the Tolerance Averaging Technique, from the point of view of the carried-out considerations, is the micro–macro decomposition assumption, according to Equation (2):

(2) $$\theta \left(x_{1},\;x_{2}\right)=\vartheta \left(x_{1},\;x_{2}\right)+g_{a}\left(x_{1},\;x_{2}\right)\cdot \psi_{a}\left(x_{1},\;x_{2}\right),$$

where the total temperature field θ is divided into an averaged part ϑ and an oscillating part in the form of product *g_a_*·ψ*_a_*. The average temperature (the so-called macrotemperature) is denoted by ϑ, the fluctuation amplitude of the temperature (the new additional unknowns) is denoted by ψ*_a_*, and the fluctuation shape functions are denoted by *g_a_*. With reference to periodic structures and structures with a functional gradation of properties, index *a* in (2) is equal to one, and the saw-type function is assumed as the *g*_1_-function. The *g*_1_-function was chosen based on the available literature and taking into account the specifics of the issue under consideration (the heat conduction issue). This function, shown in Figure 3, provides the continuity of the temperature field and the heat flux between the cells and on interfaces between the first and the second material.

**Figure 4:**
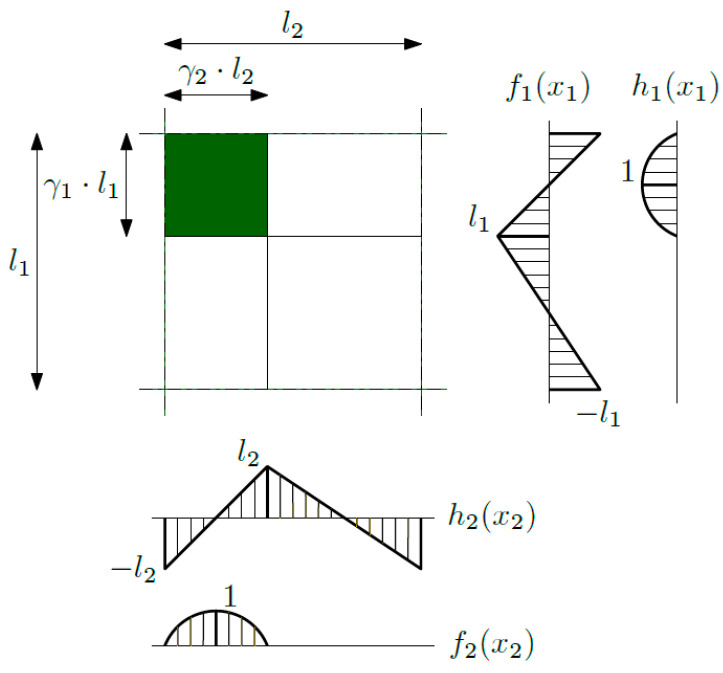
Fluctuation shape functions *g*_1_ and *g*_2_.

With reference to biperiodic structure index, *a* in (2) runs over 1 and 2. For this structure, which has the fluctuation shape functions *g*_1_ and *g*_2_, some combination of the saw-type *f_b_* and piecewise parabolic *h_b_* function was chosen, and it is shown in Figure 4. The fluctuation shape function *g*_1_(*x*_1_,*x*_2_) can be expressed as a product of functions *f*_1_(*x*_1_) and *f*_2_(*x*_2_), and the fluctuation shape function *g*_2_(*x*_1_,*x*_2_) can be expressed as a product of functions *h*_1_(*x*_1_) and *h*_2_(*x*_2_).

In addition to the micro–macro decomposition assumption, the Tolerance Averaging Technique also introduces the other assumptions and definitions such as the averaging operation, tolerance-periodic function, slowly varying function (examples include the macro-temperature function ϑ and the fluctuation amplitude ψ*_i_*) and the highly oscillating function (an example is the fluctuation shape function *g_a_*).

By using the above definitions and assumptions of the Tolerance Averaging Technique discussed in [29], the equations with the averaged coefficients describing the heat transfer phenomenon were obtained. The equations in reference to the periodic structure and the structure with a functional gradation of properties (the so-called tolerance model equations) are shown below:

(3) $$\langle c\rho \rangle \dot{\vartheta }+\langle c\rho g\rangle {\dot{\psi }}_{2}-\nabla \cdot \left(\langle K\rangle \cdot \nabla \vartheta +\langle K\cdot \partial g\rangle \psi_{2}+\langle Kg\rangle \cdot \bar{\nabla }\psi_{2}\right)=0,$$

(4) $$\begin{array}{l} \langle c\rho gg\rangle {\dot{\psi }}_{2}-\bar{\nabla }\cdot \left(\langle Kgg\rangle \cdot \bar{\nabla }\psi_{2}+\langle Kg\cdot \partial g\rangle \psi_{2}+\langle Kg\rangle \cdot \nabla \vartheta \right)+\\ +\langle K\cdot \partial g\partial g\rangle \psi_{2}+\langle Kg\cdot \partial g\rangle \cdot \bar{\nabla }\psi_{2}+\langle K\cdot \partial g\rangle \cdot \nabla \vartheta =0, \end{array}$$

where tensor, whose components are *k_ij_*, is denoted by **K,** and is the tensor of conductivity; gradient operator ∇ is expressed as follows (∂_1_,∂_2_,∂_3_); operator ∂ = (∂_1_,0,0) is a gradient in the *x*_1_-direction; and the overlined nabla operator is a gradient in the *x*_2_- and *x*_3_-directions.

The equations in reference to the biperiodic structure are shown below:

(5) $$\langle c\rho \rangle \dot{\vartheta }+\langle c\rho g_{1}\rangle {\dot{\psi }}_{1}+\langle c\rho g_{2}\rangle {\dot{\psi }}_{2}-\nabla \cdot \left(\begin{array}{l} \langle K\rangle \cdot \nabla \vartheta +\langle K\cdot \partial g_{1}\rangle \psi_{1}+\langle Kg_{1}\rangle \cdot \bar{\nabla }\psi_{1}+\\ +\langle K\cdot \partial g_{2}\rangle \psi_{2}+\langle Kg_{2}\rangle \cdot \bar{\nabla }\psi_{2} \end{array}\right)=0,$$

(6) $$\begin{array}{l} \langle c\rho g_{1}\rangle \dot{\vartheta }+\langle c\rho g_{1}g_{1}\rangle {\dot{\psi }}_{1}+\langle c\rho g_{1}g_{2}\rangle {\dot{\psi }}_{2}+\langle K\cdot \partial g_{1}\rangle \cdot \nabla \vartheta +\langle K\cdot \partial g_{1}\partial g_{1}\rangle \psi_{1}+\langle K\cdot \partial g_{1}g_{1}\rangle \cdot \bar{\nabla }\psi_{1}+\\ +\langle K\cdot \partial g_{1}\partial g_{2}\rangle \psi_{2}+\langle K\cdot \partial g_{1}g_{2}\rangle \cdot \bar{\nabla }\psi_{2}-\bar{\nabla }\cdot \left(\begin{array}{l} \langle Kg_{1}\rangle \cdot \nabla \vartheta +\langle K\cdot \partial g_{1}g_{1}\rangle \psi_{1}+\langle Kg_{1}g_{1}\rangle \cdot \bar{\nabla }\psi_{1}+\\ +\langle K\cdot \partial g_{2}g_{1}\rangle \psi_{2}+\langle Kg_{1}g_{2}\rangle \cdot \bar{\nabla }\psi_{2} \end{array}\right)=0, \end{array}$$

(7) $$\begin{array}{l} \langle c\rho g_{2}\rangle \dot{\vartheta }+\langle c\rho g_{2}g_{1}\rangle {\dot{\psi }}_{1}+\langle c\rho g_{2}g_{2}\rangle {\dot{\psi }}_{2}+\langle K\cdot \partial g_{2}\rangle \cdot \nabla \vartheta +\langle K\cdot \partial g_{1}\partial g_{2}\rangle \psi_{1}+\langle K\cdot \partial g_{2}g_{1}\rangle \cdot \bar{\nabla }\psi_{1}+\\ +\langle K\cdot \partial g_{2}\partial g_{2}\rangle \psi_{2}+\langle K\cdot \partial g_{2}g_{2}\rangle \cdot \bar{\nabla }\psi_{2}-\bar{\nabla }\cdot \left(\begin{array}{l} \langle Kg_{2}\rangle \cdot \nabla \vartheta +\langle K\cdot \partial g_{1}g_{2}\rangle \psi_{1}+\langle Kg_{1}g_{2}\rangle \cdot \bar{\nabla }\psi_{1}+\\ +\langle K\cdot \partial g_{2}g_{2}\rangle \psi_{2}+\langle Kg_{2}g_{2}\rangle \cdot \bar{\nabla }\psi_{2} \end{array}\right)=0 \end{array}$$

where operator ∂ = (∂_1_,∂_2_,0) is a gradient in the *x*_1_- and *x*_2_-directions and the overlined ∇ operator is a gradient in the *x*_3_-direction.

## 4. Finite Difference Method

It is well-known that the Finite Difference Method, when applied to the differential equation (DE), ordinary equation (ODE) or partial equation (PDE), leads to the system of algebraic linear equations, which is its biggest advantage. The domain of unknown function is narrowed down to a grid of nodes, while the codomain is replaced by set of function values in these nodes. Moreover, every derivative appearing in the DE is approximated by the finite difference quotient, depending very often on function values at adjacent nodes, and that, in particular, may cause a need to temporarily create and use virtual nodes (outside the domain). Function values at virtual nodes are acquired from boundary conditions.

What distinguishes the considered problem from the others is that we actually deal here with the system of PDEs, thus we have more than one unknown function to find. Moreover, each one may have different type of condition in common for all boundaries. Hence, most of the work that has been conducted here was grouping and ordering unknowns and constructing matrix of coefficients for the system. When everything was settled, the MAPLE environment were used in order to implement the Finite Difference Method, and that implementation is further called an algorithm.

**Figure 5:**
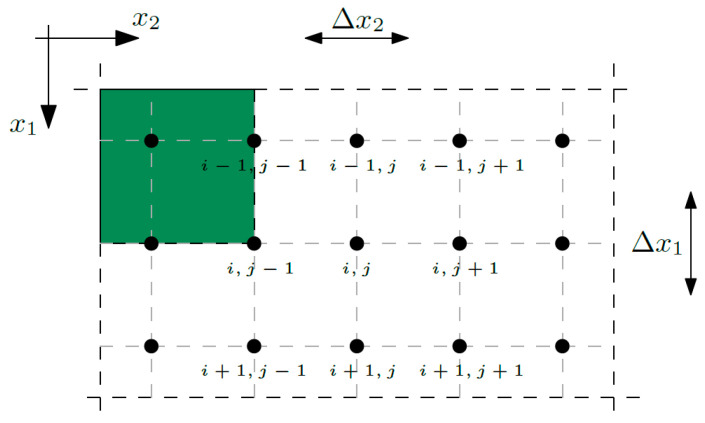
Discretized composite.

To create an algorithm of the Finite Difference Method, it is necessary to divide an analyzed structure into ranges, redefine the tolerance model equations to formulate them in index notation, replace the derivatives appearing in these equations by appropriate differential quotients, and define the boundary conditions, because this affects the number of equations to solve [26]. All of the structures, whether periodic, biperiodic, or with a functional gradation of properties, were discretized along the *x*_1_-direction into *m* ranges, so the number of the nodes along the *x*_1_-direction equals *m* + 1, and analogously along the *x*_2_-direction into *n* ranges, so the number of the nodes along the *x*_2_-direction equals *n* + 1; refer to Figure 5.

Thus, Δ*x*_1_ equals *L*_1_/*m* and Δ*x*_2_ equals *L*_2_/*n*, where *L*_1_ and *L*_2_ are the dimensions of the composite.

**Figure 6:**
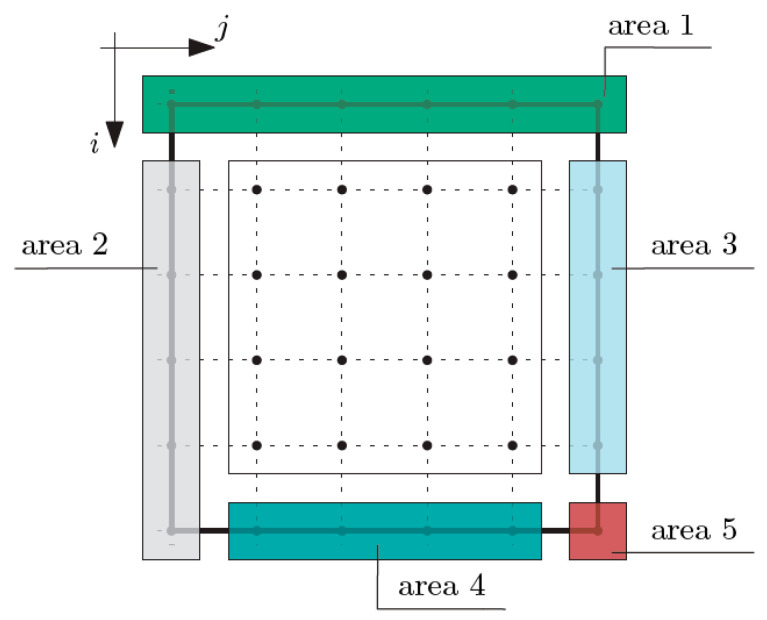
Areas for the definition of boundary conditions for macro-temperature ϑ.

Then, the thermal boundary conditions were formulated, dividing the composite into the areas shown in Figure 6.

At the nodes of area 1 (*i* equals 1 and *j* is from 1 to *n* + 1), the total temperature θ is known (defined) and hence the macro-temperature ϑ is also known at these nodes. Analogously, at the nodes of area 2 (*i* is from 2 to *m* + 1 and *j* equals 1), the macro-temperature ϑ is known. The nodes of area 3 (*i* is from 2 to *m* and *j* equals *n* + 1) and the nodes of area 4 (*i* equals *m* + 1 and *j* is from 2 to *n*) belong to the right and bottom edges of the composite, which were assumed as thermally insulated, which leads to the following conditions:

(8) $$\langle k_{11}\rangle \partial_{1}\vartheta +\langle k_{11}\partial_{1}g_{1}\rangle \psi_{1}=0,$$

(9) $$\langle k_{22}\rangle \partial_{2}\vartheta +\langle k_{22}\partial_{2}g_{2}\rangle \psi_{2}=0,$$

**Figure 7:**
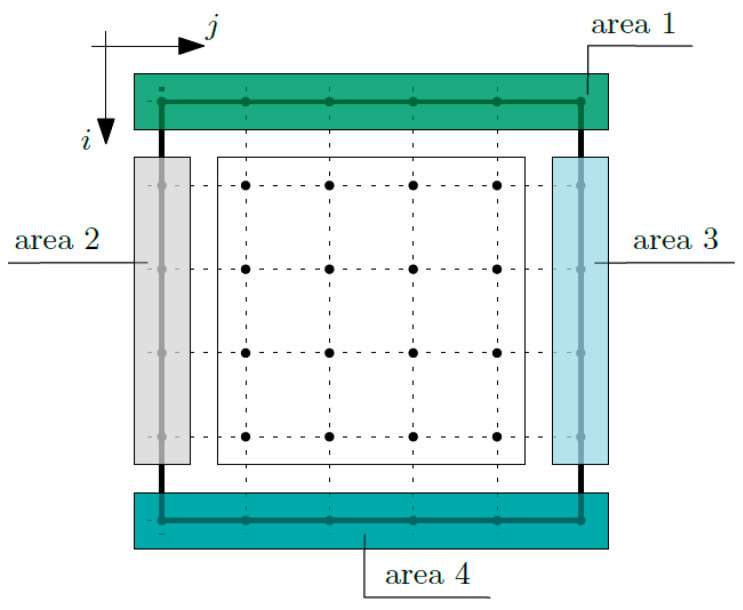
Areas for the definition of boundary conditions for fluctuation amplitudes ψ_1_ and ψ_2_.

At the nodes of area 5 (*i* equals *m* + 1 and *j* equals *n* + 1), both conditions expressed by Equations (8)–(9) are defined. Then, the boundary conditions for fluctuation amplitudes of the temperature ψ_1_ and ψ_2_ are defined, following the areas shown in Figure 7. At the nodes of area 1 (*i* equals 1 and *j* is from 1 to *n* + 1), the fluctuation amplitudes of the temperature ψ_1_ and fluctuations amplitudes of the temperature ψ_2_ are defined. Likewise, the conditions in other areas are: area 2 (*i* is from 2 to *m* and *j* equals 1), area 3 (*i* is from 2 to *m* and *j* equals *n* + 1) and area 4 (*i* equals *m* + 1 and *j* is from 1 to *n* + 1).

After assuming the boundary conditions, the matrix of coefficients appearing in the equations of the tolerance model was defined. These coefficients can be grouped in reference to the equations and individual node areas [25,26]. In an analogous way, the vector of free terms is defined. These terms result from the defined boundary conditions, which are the known values of the macro-temperature ϑ on the top and the left edges of the composite (area 1 and area 2; refer to Figure 6) and the known values of the fluctuation amplitudes of the temperature ψ_1_ and ψ_2_ on all of the edges of the composite (areas 1 to 4; refer to Figure 7).

Thus, a system of non-uniform equations was formulated. To solve this system, some variation of the Finite Difference Method was used—the so-called Crank–Nicolson method (the mixed method). The characteristic parameter for this method was assumed to be equal to a half. The Crank–Nicolson method guarantees the convergence of the solution regardless of the division of the structure.

## 5. Results

Using the created algorithm of the Finite Difference Method [23,25,26,28], calculations were conducted for the three structures described above. The two-dimensional, non-stationary heat-conduction problem of composites with base dimensions equal to *L*_1_ = *L*_2_ = 1 [m] was analyzed. During the analysis, the composite was assumed to consist of ten cells. In the case of the periodic structure, the dimension of the first sub-cell γ_2_ is taken initially as equal to 0.25; however, in the case of the structure with a functional gradation of properties, the function of gradation γ_2_(*x*_2_) equals *x*_2_/*L*_2_. On the other hand, in reference to the biperiodic structure, the volume share of the first sub-cell is originally equal to γ_1_ · γ_2_, where γ_1_ = γ_2_ = 0.25. The subsequent material properties were preliminarily established for the first sub-cell: *c* = 500 [J kg^−1^ K^−1^], ρ = 7800 [kg m^−3^], *k* = 58 [W m^−1^ K^−1^] and for the second sub-cell: *c* = 920 [J kg^−1^ K^−1^], ρ = 2720 [kg m^−3^], *k* = 200 [W m^−1^ K^−1^].

### 5.1. Periodic and Biperiodic Structure

**Figure 8:**
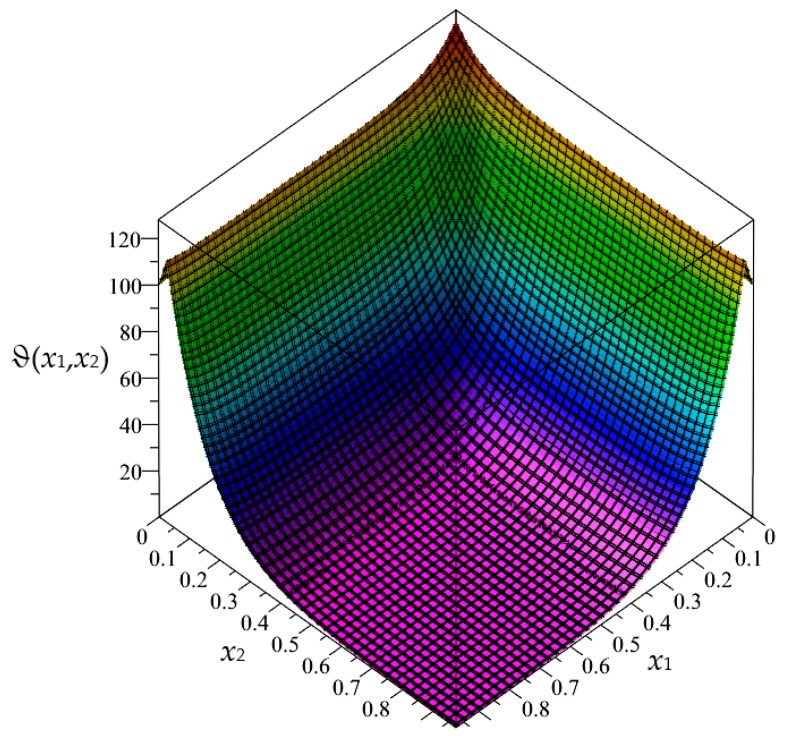
Map of the averaged temperature ϑ for biperiodic structure.

**Figure 9:**
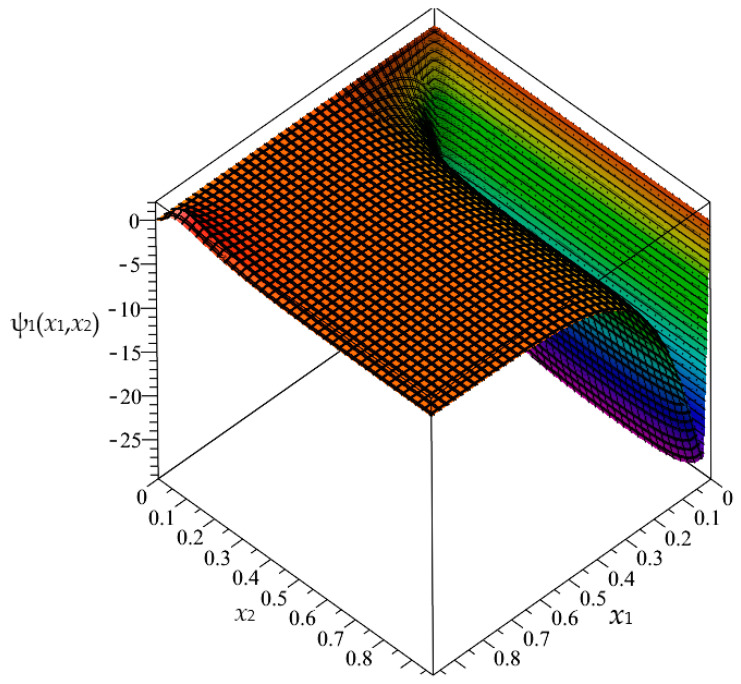
Map of the fluctuation amplitude ψ_1_ for biperiodic structure.

**Figure 10:**
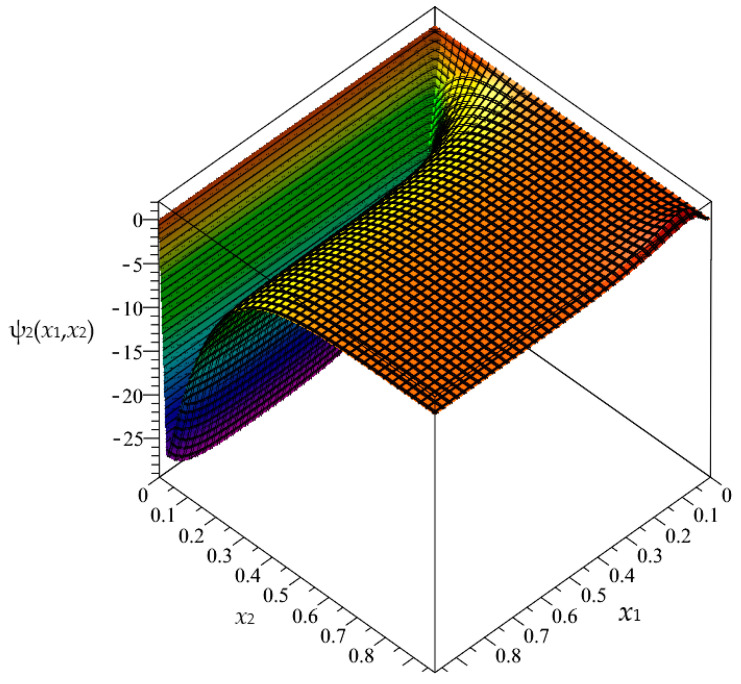
Map of the fluctuation amplitude ψ_2_ for biperiodic structure.

**Figure 11:**
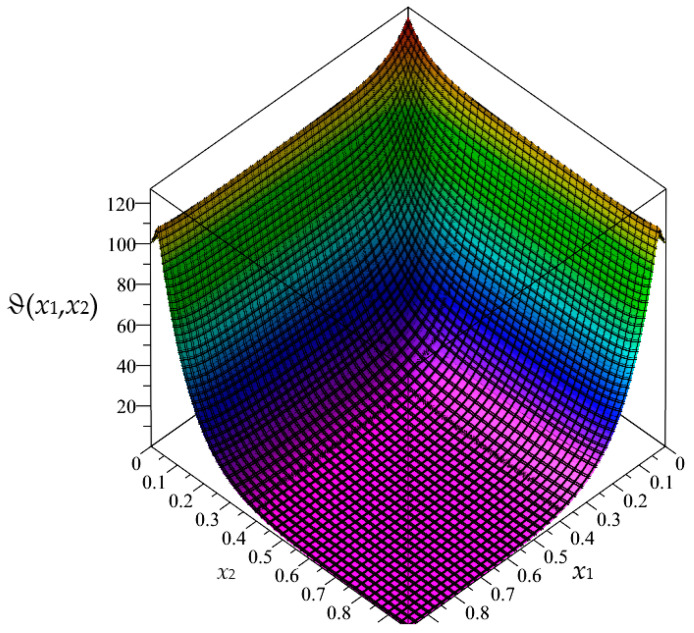
Map of the averaged temperature ϑ for periodic structure.

**Figure 12:**
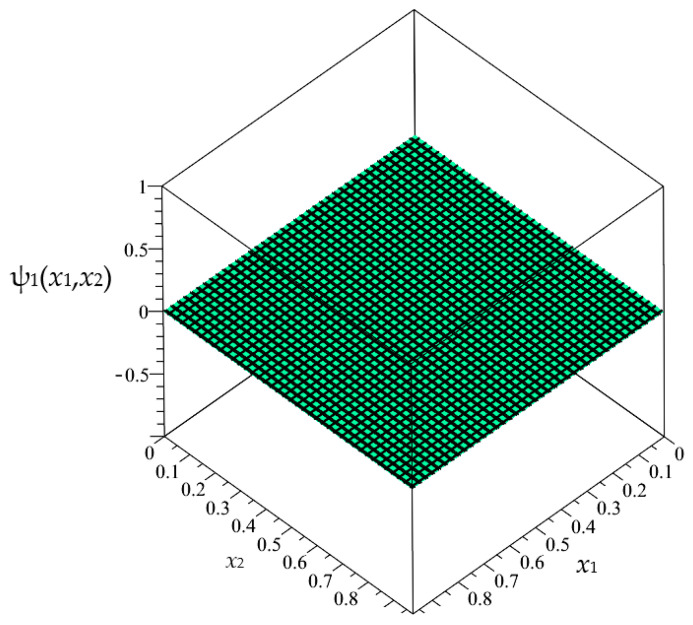
Map of the fluctuation amplitude ψ_1_ for periodic structure.

**Figure 13:**
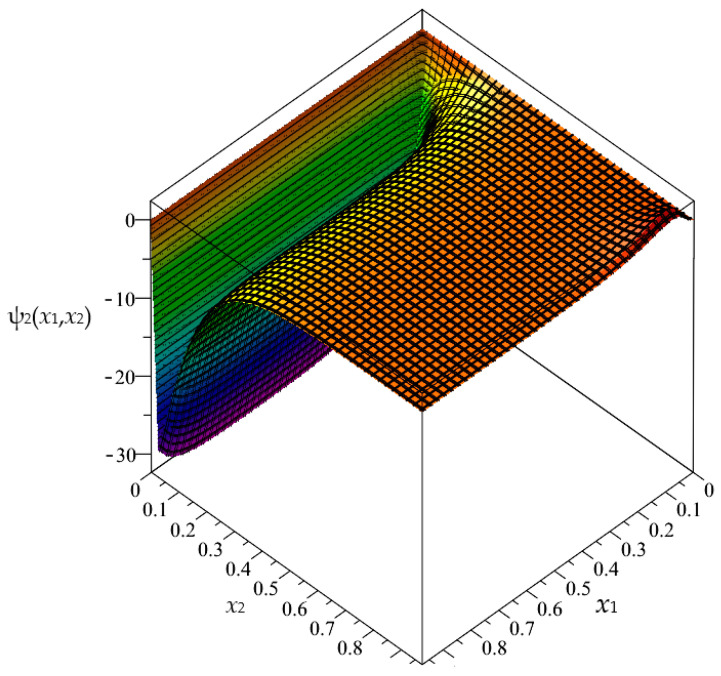
Map of the fluctuation amplitude ψ_2_ for periodic structure.

Firstly, in this section, the approximated results of the Finite Difference Method are presented in the form of 3D-maps of the averaged temperature ϑ and the fluctuation amplitudes ψ_1_ and ψ_2_; refer to Figure 8, Figure 9, Figure 10, Figure 11, Figure 12 and Figure 13.

**Figure 14:**
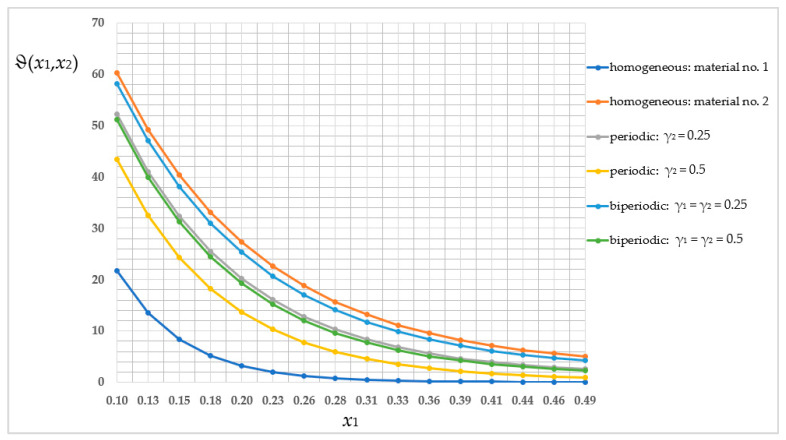
Comparison of values of the averaged temperature ϑ in selected cross-section.

Then, the average temperature distribution was compared in a homogeneous structure with properties of the first sub-cell (blue line), then the second sub-cell (orange line); the periodic structure where γ_2_ = 0.25 (grey line); the periodic structure where γ_2_ = 0.5 (yellow line); the biperiodic structure where γ_1_ = γ_2_ = 0.25 (light-blue line); and the biperiodic structure where γ_1_ = γ_2_ = 0.5 (green line). This comparison of the selected *x*_2_-coordinate (*x*_2_ = 0.5 [m]) is shown in Figure 14. For the readability of the chart, it is limited to nodes *i* from 5 to 20.

The values of the averaged temperature, shown in Figure 14, are summarized in Table 1. The lowest temperature values (ignoring the homogeneous structure) were obtained for the periodic structure where γ_2_ = 0.5, and the highest were obtained for the biperiodic structure where γ_1_ = γ_2_ = 0.25. The distinctions in values of the averaged temperature ϑ between these structures are in the order of 25.3–77.1% relative to a higher value.

**Figure 15:**
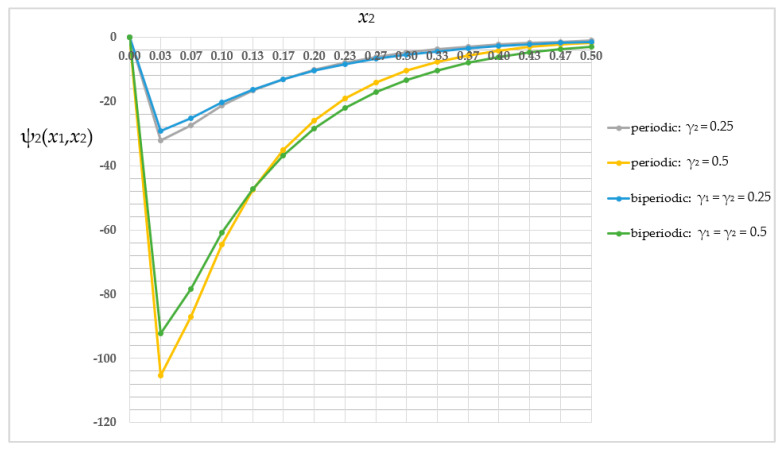
Comparison of values of the fluctuation amplitude ψ_2_ in selected cross-section.

Then, the fluctuation amplitude ψ_2_ distribution was compared (fluctuation amplitude ψ_1_ does not occur in the case of the periodic structure) in the periodic structure where γ_2_ = 0.25 (grey line); the periodic structure where γ_2_ = 0.5 (yellow line); the biperiodic structure where γ_1_ = γ_2_ = 0.25 (light-blue line); the and biperiodic structure where γ_1_ = γ_2_ = 0.5 (green line). This comparison for the selected *x*_1_-coordinate (*x*_1_ = 0.5 [m]) is shown in Figure 15. For the readability of the chart, it is limited to nodes *j* from 1 to 16.

The lowest fluctuation amplitude values (the absolute values) were obtained for the biperiodic structure where γ_1_ = γ_2_ = 0.25 and the highest were obtained for the periodic structure where γ_2_ = 0.5. The distinctions in values of the fluctuation amplitude ψ_2_ between analyzed structures are in the order of 13.65–72.21% relative to a higher value.

### 5.2. Periodic and Functionally Graded Structure

**Figure 16:**
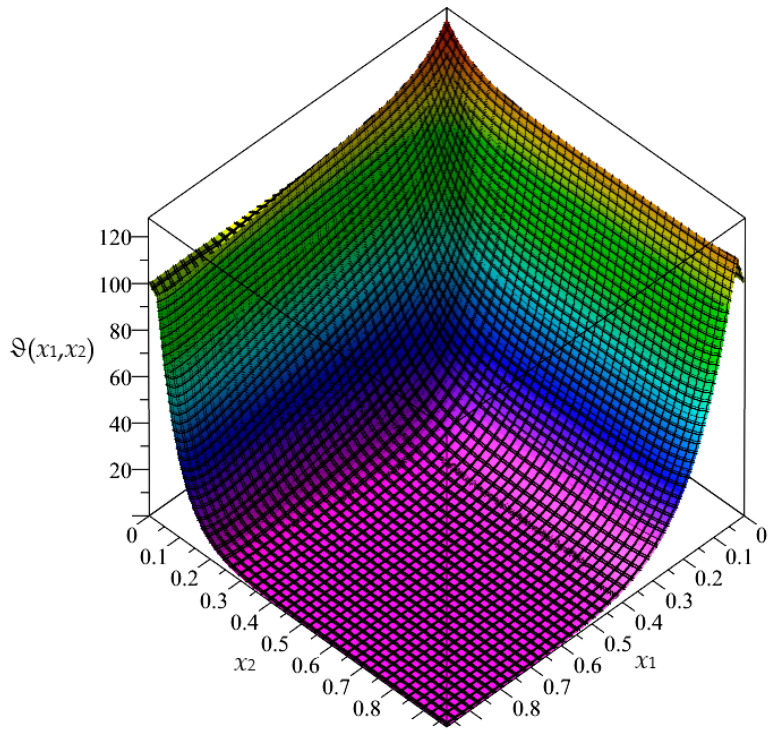
Map of the averaged temperature ϑ for functionally graded structure.

**Figure 17:**
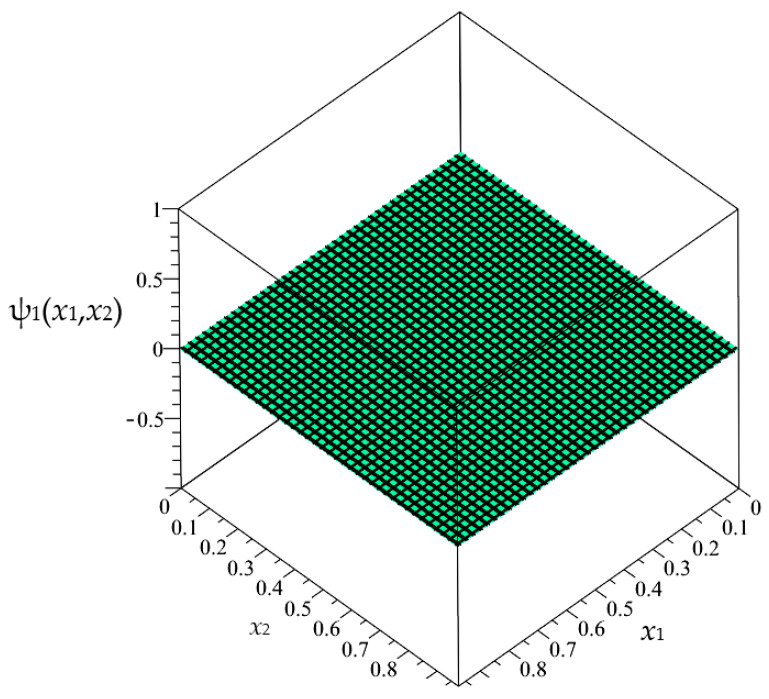
Map of the fluctuation amplitude ψ_1_ for functionally graded structure.

**Figure 18:**
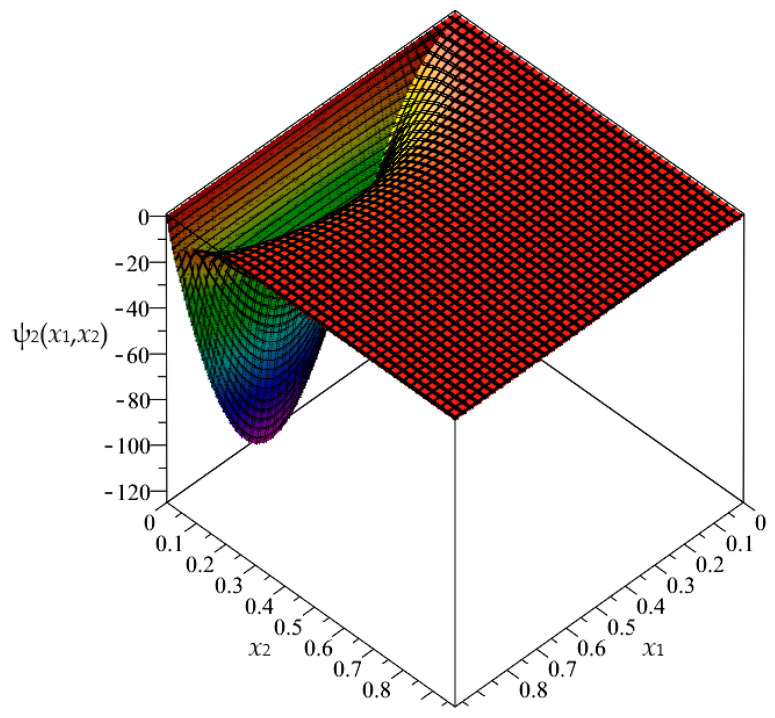
Map of the fluctuation amplitude ψ_2_ for functionally graded structure.

In this section, the approximated results of the Finite Difference Method are presented in the form of 3D-maps of the averaged temperature ϑ and fluctuation amplitudes ψ_1_ and ψ_2_ in reference to the functionally graded structure; refer to confer Figure 16, Figure 17 and Figure 18.

**Figure 19:**
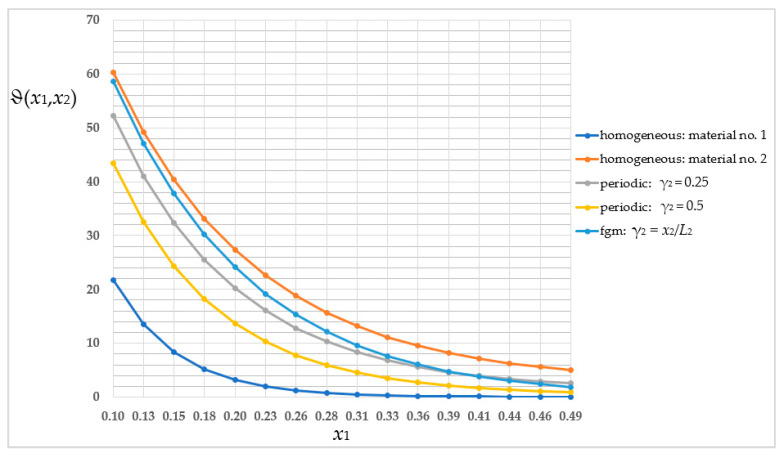
Comparison of values of the averaged temperature ϑ in selected cross-section.

Then, as in the previous section, the averaged temperature distribution was compared in a homogeneous structure with the properties of the first sub-cell (blue line) and of the second sub-cell (orange line); the periodic structure where γ_2_ = 0.25 (grey line); the periodic structure where γ_2_ = 0.5 (yellow line); and the structure with a functional gradation of properties where γ_2_ = *x*_2_/*L*_2_ (light-blue line). As before, the comparison for the selected *x*_2_-coordinate and for nodes *i* from 5 to 20 is shown in Figure 19.

The values of the averaged temperature, shown in Figure 19, are summarized in Table 2. For most of the nodes (ignoring the homogeneous structure), the highest values of the averaged temperature were observed for the functionally graded structure. The distinctions in values of the averaged temperature ϑ between the structure with a functional gradation of properties and the periodic structure where γ_2_ = 0.5 are in the order of 25.91–55.22% relative to a higher value.

**Figure 20:**
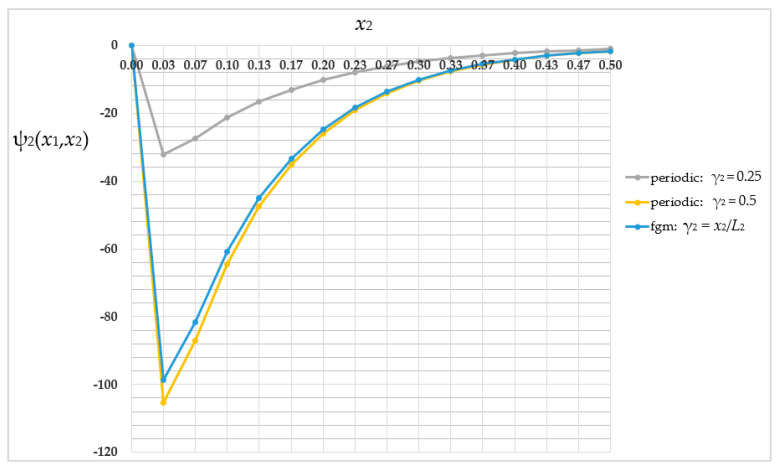
Comparison of values of the fluctuation amplitude ψ_2_ in selected cross-section.

The fluctuation amplitude ψ_2_ distribution was also compared in the periodic structure where γ_2_ = 0.25 (grey line); the periodic structure where γ_2_ = 0.5 (yellow line); and the functionally graded structure where γ_2_ = *x*_2_/*L*_2_ (light-blue line). This comparison for the selected *x*_1_-coordinate and nodes *j* from 1 to 16 is shown in Figure 20.

The lowest fluctuation amplitude values (the absolute values) were obtained for the periodic structure where γ_2_ = 0.25 and the highest were obtained for the periodic structure where γ_2_ = 0.5. The distinctions in values of the fluctuation amplitude ψ_2_ between analyzed structures are in the order of 34.88–69.41% relative to a higher value.

### 5.3. Convergence Study

For each of the considered structures, a solution convergence analysis was carried out. The analysis consisted of examining the differences in the values of the unknowns. The solution was assumed to converge when, by increasing the number of intervals Δ*x*_1_ and Δ*x*_2_, the differences in the values of the unknowns decreased and were not significant. The results shown in Section 5.1 and Section 5.2, obtained for the number of intervals Δ*x*_1_ and Δ*x*_2_, were equal to 40. 

#### 5.3.1. Biperiodic Structure

At this point, the results of a solution convergence test for the biperiodic structure are shown. In Table 3, the values of the averaged temperature ϑ(*x*_1_,*x*_2_) and the fluctuation amplitudes ψ_1_(*x*_1_,*x*_2_) and ψ_2_(*x*_1_,*x*_2_) for a selected point (*x*_1_ = *L*_1_/2, *x*_2_ = *L*_2_/2) and changing number of intervals Δ*x*_1_ and Δ*x*_2_ are summarized. In addition, the differences in values of the averaged temperature with the smallest increase in the number of intervals were calculated. These differences were denoted as Δϑ(*x*_1_,*x*_2_). Analogous differences were calculated for fluctuation amplitudes and denoted as Δψ_1_(*x*_1_,*x*_2_) and Δψ_2_(*x*_1_,*x*_2_).

As can be observed in Table 3, the differences in the values of the unknowns decrease as the number of intervals increases. The differences with the smallest increase in the number of intervals are in the order of 0.35–1.02% relative to a higher value.

#### 5.3.2. Periodic Structure

At this point, the results of a solution convergence analysis for the periodic structure are shown. In Table 4, the values of the averaged temperature ϑ(*x*_1_,*x*_2_) and the fluctuation amplitude ψ_2_(*x*_1_,*x*_2_) for a selected point (*x*_1_ = *L*_1_/2, *x*_2_ = *L*_2_/2) and changing number of intervals Δ*x*_1_ and Δ*x*_2_ are summarized. As in the case of the biperiodic structure, the differences in values of the averaged temperature Δϑ(*x*_1_,*x*_2_) and the fluctuation amplitude Δψ_2_(*x*_1_,*x*_2_) were calculated and analyzed.

The differences in the values of the unknowns decrease as the number of intervals increases, and the smallest increase in the number of intervals are in the order of 0.45–1.06% relative to a higher value.

#### 5.3.3. Functionally Graded Structure

For the functionally graded structure, the test of a solution convergence was carried out in the same way as in relation to the previous structures. In Table 5, the values of the averaged temperature ϑ(*x*_1_,*x*_2_) and the fluctuation amplitude ψ_2_(*x*_1_,*x*_2_) for a selected point (*x*_1_ = *L*_1_/2, *x*_2_ = *L*_2_/2) and changing number of intervals are summarized. The differences in values of the averaged temperature Δϑ(*x*_1_,*x*_2_) and the fluctuation amplitude Δψ_2_(*x*_1_,*x*_2_) were calculated and analyzed.

The differences with a smallest increase in the number of intervals are in the order of 0.41–1.07% relative to a higher value.

## 6. Conclusions

The main goal of this work was to analyze three types of structures—periodic, biperiodic and those with a functional gradation of properties—under specific thermal boundary conditions. All these studies were possible thanks to a self-created algorithm, the Finite Difference Method, which was implemented into MAPLE software and applied to derived averaged model equations. This work shows the influence of the structure of the composite on the temperature distribution. The dimension of the sub-cell of the composite’s cell is as important as the way and direction of placement of the components; therefore the volume shares of the materials with different properties are also important. As shown in Figure 14 and Figure 19, the values of the averaged temperature ϑ vary significantly, with relative differences in the order of up to 77%. Similarly, as shown in Figure 15 and Figure 20, the values of the fluctuation amplitude ψ_2_, which impacts in a major way on values of the total temperature field, vary substantially, with relative differences in the order of up to 72%. This leads to the conclusion that by designing the structure of the composite, the expected temperature distribution can be obtained, which in turn is important for the application of this type of construction. The analyzed structures also differ, importantly, in terms of self-weight or stiffness, which can be decisive in choosing the way and direction of the placement of the components and their volume shares.

## Figures and Tables

**Table 1:** Values of the averaged temperature [°C].

*i*	Uniform Structure First Material	Uniform Structure Second Material	Periodic Structure γ_2_ = 0.25	Periodic Structure γ_2_ = 0.5	Biperiodic Structure γ_1_ = γ_2_ = 0.25	Biperiodic Structure γ_1_ = γ_2_ = 0.5
5	21.76	60.25	52.18	43.42	58.12	51.14
6	13.49	49.26	41.04	32.48	47.05	39.93
7	8.36	40.35	32.33	24.33	38.16	31.24
8	5.19	33.15	25.53	18.25	31.03	24.49
9	3.22	27.31	20.21	13.71	25.31	19.25
10	2.00	22.59	16.06	10.33	20.73	15.19
11	1.25	18.77	12.82	7.81	17.05	12.04
12	0.78	15.68	10.28	5.93	14.10	9.59
13	0.49	13.18	8.31	4.53	11.73	7.70
14	0.31	11.16	6.77	3.49	9.84	6.23
15	0.20	9.52	5.56	2.71	8.32	5.09
16	0.13	8.21	4.62	2.13	7.11	4.21
17	0.09	7.14	3.89	1.70	6.14	3.52
18	0.06	6.28	3.32	1.38	5.36	2.99
19	0.04	5.58	2.88	1.14	4.74	2.58
20	0.03	5.02	2.53	0.97	4.24	2.27

**Table 2:** Values of the averaged temperature [°C].

*i*	Uniform Structure First Material	Uniform Structure Second Material	Periodic Structure γ_2_ = 0.25	Periodic Structure γ_2_ = 0.5	FGM Structure γ_2_ = *x*_2_/*L*_2_
5	21.76	60.25	52.18	43.42	58.62
6	13.49	49.26	41.04	32.48	47.12
7	8.36	40.35	32.33	24.33	37.78
8	5.19	33.15	25.53	18.25	30.21
9	3.22	27.31	20.21	13.71	24.11
10	2.00	22.59	16.06	10.33	19.20
11	1.25	18.77	12.82	7.81	15.26
12	0.78	15.68	10.28	5.93	12.12
13	0.49	13.18	8.31	4.53	9.60
14	0.31	11.16	6.77	3.49	7.61
15	0.20	9.52	5.56	2.71	6.02
16	0.13	8.21	4.62	2.13	4.76
17	0.09	7.14	3.89	1.70	3.77
18	0.06	6.28	3.32	1.38	2.98
19	0.04	5.58	2.88	1.14	2.37
20	0.03	5.02	2.53	0.97	1.88

**Table 3:** Values of the averaged temperature and fluctuation amplitudes for biperiodic structure.

*Number of Intervals* Δ*x*_1_ and Δ*x*_2_	ϑ(*x*_1_,*x*_2_)	Δϑ(*x*_1_,*x*_2_)	ψ_1_(*x*_1_,*x*_2_)	Δψ_1_(*x*_1_,*x*_2_)	ψ_2_(*x*_1_,*x*_2_)	Δψ_2_(*x*_1_,*x*_2_)
40	3.838	-	−0.5271	-	−0.5308	-
42	3.801	0.037	−0.5217	0.0054	−0.5257	0.0051
44	3.770	0.031	−0.5175	0.0042	−0.5216	0.0041
46	3.743	0.027	−0.5137	0.0038	−0.5180	0.0036
48	3.719	0.024	−0.5102	0.0035	−0.5148	0.0032
50	3.697	0.022	−0.5072	0.0030	−0.5119	0.0029
52	3.677	0.020	−0.5046	0.0026	−0.5092	0.0027
54	3.659	0.018	−0.5021	0.0025	−0.5068	0.0024
56	3.642	0.017	−0.4998	0.0023	−0.5046	0.0022
58	3.627	0.015	−0.4976	0.0022	−0.5026	0.0020
60	3.613	0.014	−0.4955	0.0021	−0.5008	0.0018

**Table 4:** Values of the averaged temperature and fluctuation amplitudes for periodic structure.

*Number of Intervals* Δ*x*_1_ and Δ*x*_2_	ϑ(*x*_1_,*x*_2_)	Δϑ(*x*_1_,*x*_2_)	ψ_2_(*x*_1_,*x*_2_)	Δψ_2_(*x*_1_,*x*_2_)
40	2.261	-	−0.3533	-
42	2.237	0.024	−0.3496	0.0037
44	2.216	0.021	−0.3463	0.0033
46	2.197	0.019	−0.3434	0.0029
48	2.180	0.017	−0.3408	0.0026
50	2.165	0.015	−0.3385	0.0023
52	2.151	0.014	−0.3364	0.0021
54	2.139	0.012	−0.3345	0.0019
56	2.128	0.011	−0.3328	0.0017
58	2.118	0.010	−0.3312	0.0016
60	2.109	0.009	−0.3297	0.0015

**Table 5:** Values of the averaged temperature and fluctuation amplitudes for FGM structure.

*Number of Intervals* Δ*x*_1_ and Δ*x*_2_	ϑ(*x*_1_,*x*_2_)	Δϑ(*x*_1_,*x*_2_)	ψ_2_(*x*_1_,*x*_2_)	Δψ_2_(*x*_1_,*x*_2_)
40	2.157	-	−0.8139	-
42	2.134	0.023	−0.8059	0.0080
44	2.113	0.021	−0.7988	0.0071
46	2.095	0.018	−0.7925	0.0063
48	2.079	0.016	−0.7869	0.0056
50	2.064	0.015	−0.7818	0.0051
52	2.051	0.013	−0.7772	0.0046
54	2.039	0.012	−0.7731	0.0041
56	2.028	0.011	−0.7693	0.0038
58	2.018	0.010	−0.7659	0.0034
60	2.009	0.009	−0.7627	0.0032

## Data Availability

Not applicable.
